# Circulating tumor cells, disease recurrence and survival in newly diagnosed breast cancer

**DOI:** 10.1186/bcr3333

**Published:** 2012-10-22

**Authors:** Bas Franken, Marco R de Groot, Walter JB Mastboom, Istvan Vermes, Job van der Palen, Arjan GJ Tibbe, Leon WMM Terstappen

**Affiliations:** 1 Department of Internal Medicine, Medisch Spectrum Twente, Haaksbergerstraat 55, Enschede, 7513 ER, The Netherlands; 2 Department of Surgery, Medisch Spectrum Twente, Haaksbergerstraat 55, Enschede, 7513 ER, The Netherlands; 3 Department of Clinical Chemistry, Medisch Spectrum Twente, Haaksbergerstraat 55, Enschede, 7513 ER, The Netherlands; 4 Department of Epidemiology, Medisch Spectrum Twente, Haaksbergerstraat 55, Enschede, 7513 ER, The Netherlands; 5 Department of Research Methodology, Measurement and Data Analysis, University of Twente, Drienerlolaan 5 Enschede, 7522 NB, The Netherlands; 6 VyCAP, Abraham Rademakerstraat 41, Deventer, 7425 PG, The Netherlands; 7 Medical Cell BioPhysics group, MIRA Institute, University of Twente, Drienerlolaan 5, Enschede, 7522 NB, The Netherlands

## Abstract

**Introduction:**

The presence of circulating tumor cells (CTC) is an independent prognostic factor for progression-free survival and breast cancer-related death (BRD) for patients with metastatic breast cancer beginning a new line of systemic therapy. The current study was undertaken to explore whether the presence of CTC at the time of diagnosis was associated with recurrence-free survival (RFS) and BRD.

**Methods:**

In a prospective single center study, CTC were enumerated with the CellSearch system in 30 ml of peripheral blood of 602 patients before undergoing surgery for breast cancer. There were 97 patients with a benign tumor, 101 did not meet the inclusion criteria of which there were 48 patients with DCIS, leaving 404 stage I to III patients. Patients were stratified into unfavorable (CTC ≥1) and favorable (CTC = 0) prognostic groups.

**Results:**

≥1 CTC in 30 ml blood was detected in 15 (15%) benign tumors, in 9 DCIS (19%), in 28 (16%) stage I, 32 (18%) stage II and in 16 (31%) patients with stage III. In stage I to III patients 76 (19%) had ≥1 CTC of whom 16 (21.1%) developed a recurrence. In 328 patients with 0 CTC 38 (11.6%) developed a recurrence. Four-year RFS was 88.4% for favorable CTC and 78.9% for unfavorable CTC (*P *= 0.038). A total of 25 patients died of breast cancer-related causes and 11 (44%) had ≥1 CTC. BRD was 4.3% for favorable and 14.5% for unfavorable CTC (*P *= 0.001). In multivariate analysis ≥1 CTC was associated with distant disease-free survival, but not for overall recurrence-free survival. CTC, progesterone receptor and N-stage were independent predictors of BRD in multivariate analysis.

**Conclusions:**

Presence of CTC in breast cancer patients before undergoing surgery with curative intent is associated with an increased risk for breast cancer-related death.

## Introduction

With 1.15 million new cases each year, breast cancer is the most common form of cancer among women worldwide [1]. Patients with non-metastatic breast cancer are treated surgically with or without adjuvant therapy. Adjuvant treatments are only indicated if they significantly reduce the risk for recurrence. Risk assessment is of utmost importance because of the well-known side effects of adjuvant treatment and is being conducted by means of TNM classification and differentiation grade complemented by estrogen and progesterone receptor status, Her2neu expression and peritumoral vascular invasion [2-6]. More recent improvement of the risk assessment is obtained through the molecular characterization of the tumor and identifies patients who are predicted to obtain the most therapeutic benefit [7-15]. These methods identify genetic phenotypes with a higher likelihood for micrometastasis that can lead to disease recurrence. Detection of the actual presence of tumor cells beyond the primary tumor is preferred, but may not be sufficient, as one cannot distinguish between dormant tumor cells and those giving rise to recurrence of the disease [16,17]. The presence of micrometastases in bone marrow of breast cancer patients is associated with an increased risk for disease recurrence and death [18,19]. Detection of bone marrow micrometastasis has, however, not been adapted as standard in clinical practice [20]. A more attractive approach for the detection of the presence of tumor cells beyond the primary tumor is the detection of circulating tumor cells (CTC). For CTC detection a validated method is available [21] and several studies have demonstrated that the presence of CTC in patients with metastatic breast cancer is associated with a significantly shorter progression-free and overall survival [22-29]. In these studies, CTC can be found in approximately 70% of metastatic breast cancer patients. Before receiving neoadjuvant therapy for breast cancer the frequency in which CTC are detected is significantly lower (approximately 20%), and their presence was associated with a significant risk for recurrence [30]. The purpose of this study is to examine the frequency of CTC in non-metastatic breast cancer and determine whether their presence is associated with an increased recurrence rate or breast cancer-related death (BRD).

## Materials and methods

### Study design

In this single-blind prospective study, 602 patients were enrolled before surgery for a breast tumor with curative intend. To increase the sensitivity, 30 ml of blood was drawn in all patients into four CellSave preservative tubes (Veridex, Raritan, NJ, USA) before surgery to measure CTC. Patients who were 18 years or older, had an ECOG performance status of 0 or 1, and clinical stage I to III breast cancer were included in the study. Patients who were found to have a benign tumor after histological analysis of the removed tumor were placed in a control group. Patients with the following characteristics were excluded: ductal carcinoma *in situ *(DCIS), other malignancies at the time of inclusion or within five years prior to inclusion except skin malignancy other than melanoma or cervical carcinoma *in situ*, patients with stage IV breast cancer at inclusion, male breast cancer. All patients were treated in accordance with Dutch national guidelines [31]. According to these guidelines, adjuvant systemic treatment (hormonal therapy or chemotherapy) is indicated in node-positive patients, node-negative patients younger than 35 except those with a grade I tumor ≤ cm, patients of 35 years and older with a tumor larger than 1 cm and patients older than 35 except those with a grade I (and grade II until 2010) between 1 and 2 cm. Chemotherapy was advised up to 70 years old followed by hormonal therapy. Adjuvant Online [32] was used to calculate survival increase due to chemotherapy and, if this was found insignificant, chemotherapy was omitted. During the study, the chemotherapy regimen was changed from anthraclines-based to regimens that also included taxanes. Trastuzumab treatment was given for one year. There was no difference in treatment between CTC-positive and CTC-negative patients since treating physicians were blinded for CTC results. Follow-up was also in accordance with these guidelines: every three months in the first year with a yearly mammogram, every six months in the second year with a yearly mammogram, once a year from three to five years with yearly mammogram. After five years, all patients younger than 60 were seen yearly with a mammogram. For patients older than 60 this was once every two years. The ethics board of Medisch Spectrum Twente, Enschede, The Netherlands approved the study protocol and all patients provided informed consent. We expected that 25% of patients would have CTC and a recurrence rate of 10% in the CTC-negative group and 20% in the CTC-positive group. For 80% power we calculated a sample size of 500 patients. A minimal follow-up period of six months was chosen. Body Mass Index was not captured and thus not considered in our analysis.

### CellSearch system

The CellSearch system (Veridex, Raritan, NJ, USA) was used to measure CTC. Four 7.5 ml aliquots of each patient were analyzed within 72 hours after blood draw. This system immunomagnetically enriches CTC from 7.5 ml of blood targeting the epithelial cell adhesion molecules (EpCAM). The enriched cells are labeled with the nucleic acid dye 4',6-diaminodino-2-phenylindole (DAPI) and antibodies specific for leukocytes (CD45) labeled with allophycocyan (APC) and specific for epithelial cells (cytokeratin 8, 18, 19) labeled with phycoerythrin (PE). Images of CTC candidates were captured by a semi-automatic magnetic fluorescence microscope and presented to experienced operators for classification as CTC when the cells were larger than 4 μm, expressed cytokeratin and lacked CD45 [21]. The operators were blinded to the clinical status of the patient.

### Statistical analysis

Two databases were created, one with the results of the CTC analysis, patient ID and inclusion date and one with the clinical data from the patient charts; both were merged at the time of the analysis at the hospital. The following clinical data were included: age, menopausal status, tumor stage on basis of pTNM classification, estrogen/progesterone receptor (ER/PR) status, Her2Neu receptor status, differential grade of the tumor based on the Bloom-Richardson method, adjuvant treatment, date of recurrence if occurred, location of recurrence (local versus distant), date of breast cancer-associated death or non-breast cancer-associated death. ER and PR positivity was defined at 10% or more. Her2Neu positivity was defined as 3+ of 2+ with confirmation. The time until recurrence was defined as the time between date of inclusion and the date on which the recurrence was objectified with an appropriate diagnostic test. Follow-up time was defined as the time between inclusion date and the date of the last checkup. Patients with no objectified recurrence at the end of follow-up were considered free of recurrence. SPSS version 17 (SPSS Inc., Somers, NY, USA) was used for statistical analysis. Factors that have influence on recurrence, distant disease-free survival and breast cancer-related survival, were identified and placed in a multivariate logistic regression model to identify independent predictors of recurrence, distant recurrence and breast cancer-related death. In these models, all univariate significant variables were included and after step-by-step elimination of the least significant variable while observing less than 10% change in regression coefficient, a final model was reached for both recurrence and breast cancer-related death. Kaplan-Meier curves were generated for recurrence-free survival (RFS) and breast cancer-related death. The log-rank test was used to compare patients with and without CTC. For categorical variables the chi-square test was used. The Mann-Whitney *U *test was used to compare continuous variables. A *P *value smaller then 0.05 was used to indicate a significant difference. All tests are two-sided.

## Results

### Patient characteristics

Six hundred and two patients were recruited for the study between September 2003 and January 2009. This included 404 patients with stage I to III breast cancer. Their age ranged from 29 to 90 years (mean and median of 59). Ninety-seven were found to have a benign breast tumor and were placed in a control group. One hundred and one patients were excluded; 48 with DCIS, 42 due to insufficient data, 5 with unreliable CTC measurements, 4 patients turned out to have stage IV breast cancer at inclusion, 1 patient was male, and in 1 patient there was no known primary tumor. Demographics and baseline characteristics of the included patients are shown in Table 1. A total of 84.7% of patients were ER-positive and 71.8% were PR-positive, but only 47.5% of patients received adjuvant hormonal therapy in accordance with Dutch national guidelines, which shows that our study includes a large group of patients with relative favorable characteristics. Twenty-seven of 81 Her2 positive-patients received adjuvant treatment with trastuzumab. Adjuvant treatment with trastuzumab was started at our hospital at the end of 2005.

**Table 1:** Patient characteristics, demographics and relation to recurrence-free survival and breast cancer-related death.

	n	%	RFS^1 ^(*P*)	BRD^2 ^(*P*)		n	%	RFS (*P*)	BRD (*P*)
**T stage**			0.003	0.001	**Ad chemo TX**^4^			0.201	0.759
T1	229	56.7			Yes	150	37.1		
T2	155	38.4			No	254	62.9		
	
T3	12	3.0			**Ad rad TX**^5^			0.851	0.906
T4	8	2.0			Yes	295	73.0		
	
**N stage**			0.036	0.001	No	109	27.0		
	
N0	261	64.6			**Ad hor TX**^6^			0.921	0.644
N1	99	24.5			Yes	192	47.5		
N2	42	10.4			No	212	52.5		
	
N3	2	0.5			**Age**	Continuous	0.905	0.335	

**Stage**			0.008	0.001	**Menopausal status**			0.541	0.773
I	178	44.1			Pre	119	29.5		
IIA	122	30.2			Post	212	52.5		
	
IIB	53	12.3			**CTC**			0.029	0.001
IIIA	40	10.4			Yes	76	18.8		
IIIB	9	1.7			No	328	81.2		
	
IIIC	2	0.5			**Recurrence**				
	
**Histology**			0.933	0.440	Yes	54	13.4		
Lobular	44	10.9			No	350	86.6		
	
Ductal	341	84.4			**Mortality**				
Other	19	4.7			Yes	34	8.4		
	
**Differentiation**			<0.001	<0.001	No	370	19.6		
	
I	98	24.3			**BC mortality**^7^				
II	185	45.8			Yes	25	6.2		
III	121	30.0			No	379	93.8		

**ER**^1^			0.029	<0.001	**Follow-up**				
Pos	342	84.7			min	6 months			
Neg	62	15.3			max	90 months			
	
**PR**^2^			0.061	0.001	mean	48.6 months			
Pos	290	71.8			median	48 months			
	
Neg	114	28.2							
	
**Her2/neu**^3^			0.024	0.010					
Pos	81	20.0							
Neg	323	80.0							

^1^Estrogen receptor status, ^2^progestrone receptor status, ^3^Her2/neu receptor status, ^4^adjuvant chemotherapy, ^5^adjuvant radiation therapy, ^6^adjuvant hormonal therapy, ^7^breast cancer- related mortality. BRD, breast cancer-related death; CTC, circulating tumor cells; RFS, recurrence-free survival.

### CTC

The prevalence of CTC in the patients enrolled in the study is shown in Table 2. Patients are grouped as those that met the inclusion criteria, that is stage I, II, or III disease, those with benign disease that served as the control group and the patients with DCIS that were excluded from any further analysis. In addition, the prevalence of CTC in patients with stage I, II, and III disease are provided separately.

**Table 2:** Prevalence of circulating tumor cells.

	Stage I-III		Stage I		Stage II		Stage III		DCIS		Benign	
	*n *= 404		*n *= 178		*n *= 175		*n *= 51		*n *= 48		*n *= 97	
**CTC**	**n**	**%**	**n**	**%**	**n**	**%**	**n**	**%**	**n**	**%**	**n**	**%**

0	328	81.2	150	84.3	143	81.7	35	68.6	39	81.3	82	84.5
>1	76	18.8	28	15.7	32	18.2	16	31.3	9	18.7	15	15.4
												
1	48	11.9	20	11.2	22	12.6	6	11.8	3	6.3	12	12.4
2	5	1.2	1	0.6	3	0.6	1	2.0	2	4.2	1	1.0
3	6	1.5	2	1.1	1	0.6	3	5.9	0	0	0	0
4	5	1.2	1	0.6	1	0.6	3	5.9	1	2.1	0	0
>4	12	3.0	4	2.2	5	2.9	3	5.9	3	6.3	2	2.1

CTC, circulating tumor cells; DCIS, ductal carcinoma *in situ*.

### Association between presence of CTC, disease recurrence and survival

**Figure 1:**
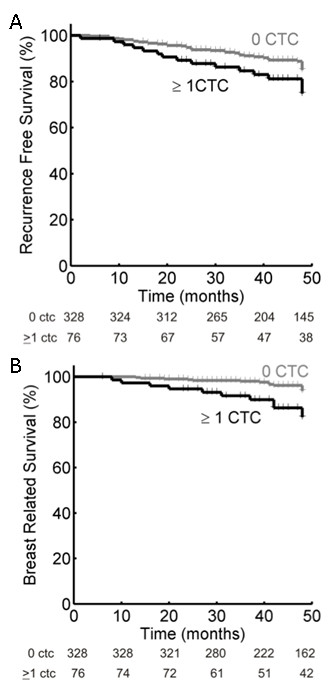
**Kaplan-Meier curves of recurrence-free survival (Panel A) and breast cancer-related death (Panel B) of 404 patients with stage I, II and III breast cancer**. Patients were divided into those with 0 circulating tumor cells (CTC) detected in 30 ml of blood and those with 1 or more CTC in 30 ml of blood.

A total of 54 patients developed disease recurrence during follow-up. Of the 76 patients with CTC, 16 (21.1%) developed a recurrence compared to 38 of 328 (11.6%) patients without CTC (*P *= 0.029). The four-year RFS is shown in Figure 1A, patients without CTC had a RFS of 88.4% and for patients with CTC this was 78.9% (log-rank *P *= 0.038). Time to recurrence ranged from 6 to 90 months. Eleven patients had a recurrence within one year of inclusion. In accordance with Dutch National Guidelines a chest X-ray or computed tomography (CT) scan, ultrasound of the liver and bone scan were performed in patients with stage III breast cancer prior to surgery, but not in patients with stage I and II breast cancer. During follow-up, a total of 34 patients died from which 25 died of breast cancer-related causes. The nine patients that died of other causes were not included in survival analysis, so that only breast cancer-related death is analyzed. Eleven of the 25 patients (40%) who died of breast cancer-related causes had CTC (44%) in contrast to 65 of the 328 patients (20%) with no CTC (log-rank *P *= 0.001). BRD is shown in Figure 1B. Of the patients without CTC, 4.3% died due to breast cancer-related causes. In the group of patients with CTC this was 14.5% (log-rank *P *= 0.001). Time till death ranged from 6 to 89 months. All 25 patients who died were also included in the recurrence group.

### Univariate analysis for disease recurrence and survival

The significance of stage, histology, differentiation, menopausal status, age, ER, PR and Her2/neu status, adjuvant therapy and CTC for RFS and BRD is shown in Table 1. T-stage, N-stage, overall stage, differential grade, ER status, PR status, Her2neu status and CTC were significant for RFS and BRD. For CTC a cutoff value of 1 or more CTC was chosen. If similar analyses are performed with higher cutoff values than CTC is no longer significant. This is probably due to the small number of patients that had 2 or more CTC.

### Multivariate analysis for disease recurrence and survival

A multivariate analysis was performed for RFS in which all univariate significant factors were included (Table 1). After stepwise exclusion of the non-significant variables the final model is shown in Table 3. This shows that T-stage and differential grade are independent prognostic factors for recurrence, but that preoperative presence of CTC is not (*P *= 0.155). However, if patients with local recurrence are excluded and only patients with distant recurrence are included in the analysis (n = 41), then preoperative presence of CTC is an independent prognostic factor (*P *= 0.015) (Table 4). For BRD the final model is shown in Table 5. This shows that CTC together with a negative progesterone receptor status and N-stage are independent predictors for death due to breast cancer-related causes.

**Table 3:** Final model of multivariate Cox regression analysis for prediction of recurrence-free survival among univariately significant parameters.

Parameter	HR	95% CI	*P*
CTC 0 vs ≥1	1.63	0.83-3.19	0.155
Differentiation grade			
I	1.00		0.003
II	2.56	0.84-7.78	0.097
III	5.49	1.82-16.54	0.003
T stage			
1	1.00		0.038
2	1.88	1.00-3.55	0.050
3+4	3.38	1.14-10.00	0.028

CI, confidence interval; CTC, circulating tumor cells; HR, hazard ratio.

**Table 4:** Final model of multivariate Cox regression analysis for prediction of distant disease-free survival among univariately significant parameters.

Parameter	HR	95% CI	*P*
CTC 0 vs ≥1	2.56	1.20-5.46	0.015
ER pos vs neg	3.71	1.72-8.06	0.010
T stage			
1 2 3+4	1.00 2.02 4.24	0.94-4.37 1.22-14.77	0.050 0.072 0.023
N stage			
0 1 2+3	1.00 3.18 2.58	1.43-7.09 0.94-7.07	0.014 0.005 0.065

CI, confidence interval; CTC, circulating tumor cells; ER, estrogen receptor; HR, hazard ratio.

**Table 5:** Final model of multivariate Cox regression analysis for prediction of breast cancer-related death.

Parameter	HR	95% CI	*P*
CTC 0 vs ≥1	3.47	1.36-8.83	0.009
PR neg vs pos	3.11	1.28-7.56	0.012
N stage			
0	1.00		0.001
1	6.83	2.529-18.463	<0.001
2+3	3.19	0.908-11.229	0.070

CI, confidence interval; CTC, circulating tumor cells; HR, hazard ratio; PR, progesterone receptor.

## Discussion

Improvement of the success rate of cancer therapy requires accurate selection of patients that will benefit from certain therapies. However, despite the array of biomarkers some low-risk patients will die from distant metastasis, whereas some high-risk patients will survive for decades. Although risk profile based on biomarkers can help to improve the ability to discriminate between low- and high-risk patients, accurate detection whether or not the tumor has actually already disseminated would be preferred. Micrometastasis in the bone marrow at diagnosis is detected in approximately 30% of breast cancer patients, their presence is independent of disease stage and is associated with an increased risk for disease recurrence [18,19]. Screening for occult metastatic tumor cells in the bone marrow has, however, not been included as standard clinical routine [20]. Reasons are that it is regarded as too invasive and no validated methods are available to reliably enumerate disseminated tumor cells routinely in clinical laboratories. Detection of tumor cells in the blood is an attractive alternative as a validated method is available and the presence of CTC in breast cancer patients with metastatic disease has shown to be an independent prognostic factor for progression-free and overall survival [22,28,29]. The frequency of CTC is, however, low and only 52% of patients starting first-line chemotherapy have 5 or more CTC in 7.5 ml of blood [23]. In patients prior to pre-neoadjuvant chemotherapy and definitive surgery the frequency is even lower and 1 or more CTC were detected in 7.5 ml of blood in 23 to 24% of these patients [29,33]. In the ongoing German SUCCESS study, 21% of patients at primary diagnosis of breast cancer, 1 or more CTC were detected in 23 ml of blood [34,35]. In this study, four 7.5 ml aliquots of blood from four blood collection tubes were investigated for the presence of CTC to increase the sensitivity of CTC detection with the FDA-cleared CellSearch CTC test. The fourfold increase in blood volume was chosen as a lesser increase in blood volume was not likely to provide sufficient benefit [36]. A recent study suggest that this blood volume is still not sufficient to detect intact nucleated, EpCAM+, cytokeratin 8,18 or 19+, CD45 CTC in all patients suggesting the need for 'CTC' apheresis or *in vivo *detection of CTC [37]. One or more CTC in 30 ml of blood were detected in 76 of the 404 (19%) patients with stage I, II, or III breast cancer. Of the 602 patients undergoing breast surgery, 48 were diagnosed with DCIS and surprisingly the proportion of DCIS patients in which CTC were detected was the same (19%) as in stage I and II cancer. The percentage of patients with detectable CTC was lower (15%) in the 97 patients with benign disease. In the control group from the original studies in metastatic breast cancer patients, 8% of patients with benign breast tumors and 4% of healthy females had 1 or more CTC in 7.5 ml of blood [21,22]. These observations are in line with the observations in this study and raise the question why a higher CTC background is observed in patients with benign disease as compared to healthy controls. Still for the identification of patients with cancer the specificity of the CTC test at this low number needs to be improved. Some improvement can be obtained by elimination of the variation between operators in assigning the identified objects as CTC [35]. Although automation of the CTC assignment can eliminate this error, it does not imply that the correct CTC definition is used [38]. Further confirmation will be needed to confirm that the events identified as CTC at this low frequency are indeed tumor cells. Technology to detect genetic abnormalities associated with cancer in CTC detected by the CellSearch system has recently been demonstrated [39]. Even though a background was observed in the control group, the presence of CTC in 30 ml of preoperatively drawn blood of patients with non-metastatic breast cancer was associated with a higher risk of recurrence and breast cancer-related death as compared to patients without CTC. In multivariate logistic regression CTC were significant for death due to breast cancer-related causes, and for distant disease-free survival, but not for overall recurrence-free survival. It has to be said that patients with CTC had higher T- and N-stages than people without CTC and therefore a larger group of CTC-positive patients than CTC-negative patients received adjuvant systemic therapy. This will probably have lowered the number of recurrences and deaths in the CTC-positive group more than in the CTC-negative group. Furthermore, of all 54 patients with recurrence, 13 had a local recurrence. The observation that presence of CTC was only significant for patients with distant recurrences suggests that it is unlikely that systemic CTCs are detected in patients with local recurrences. Our study is slightly underpowered since we needed 500 patients for a power of 80% but, in the end, only included 404 patients with invasive breast cancer. Inclusion was stopped due to cessation of funding in 2009. In this study, disease recurrence was observed in 11.6% of patients in whom no CTC were detected in 30 ml of blood. All patients with recurrent disease must have had tumor cells in the blood at some time to seed the distant metastasis and considerable improvement of the sensitivity and specificity of CTC detection will be needed to detect CTC in all patients at risk for recurrence [37]. In patients in which CTC were detected, 21.1% developed a recurrence during the follow-up period. The median follow-up was 48 months and a longer follow-up may have increased this percentage. This, however, will not obliterate the specificity issue, as it is clear that the specificity of the CTC assay will need to be increased to assure that the detected objects are indeed cancer cells. The specificity of CTC detection was brought to light by the present study through the inclusion of patients with DCIS and benign breast disease in a completely blinded fashion in contrast to other CTC studies using the CellSearch system for the enumeration of CTC in the early disease setting in which no such controls were included [29,33-35].

Moreover, one would like to discriminate between dormant, viable and tumor cells that have metastatic potential. Larger blood volumes, alternative phenotypes, additional criteria and increase in recovery of CTC are all avenues that can be pursuit to increase the sensitivity of CTC detection.

## Conclusions

Presence of CTC in breast cancer patients before undergoing surgery with curative intent is associated with an increased risk for breast cancer-related death and distant disease-free survival, but not for overall recurrence-free survival.

## Abbreviations

APC: allophycocyan; BRD: breast cancer-related death; CTC: circulating tumor cells; DAPI: 4',6-diaminodino-2-phenylindole; DCIS: ductal carcinoma *in situ*; EpCAM: epithelial cell adhesion molecules; ER: estrogen receptor; PE: phycoerythrin; PR: progesterone receptor; RFS: recurrence-free survival.

## Competing interests

This work was supported by Immunicon Corporation, responsible for the development of the CellSearch system. Prof. Leon WMM Terstappen is an inventor of several patents related to the CTC technology that have been assigned to Veridex LLC, he is presently a consultant for Veridex and receives research funding from Veridex LLC. All remaining authors have declared no conflict of interest.

## Authors' contributions

FB retrieved the patient data from the records, performed statistical analysis and helped to draft the manuscript. JP helped with the statistical analysis and drafting of the manuscript, AT was responsible for the CTC data collection and data analysis, IV was responsible for the patient blood collection and participated in the design of the study, MG, WM and LT participated in the design of the study and helped to draft the manuscript. All authors read and approved the final manuscript.
